# Reviewers

**DOI:** 10.1177/2041669516686174

**Published:** 2016-12-01

Perception and i-Perception would like to thank the following for reviewing in 2015–2016:

Adams, Wendy

Adolph, Dirk

Agostini, Tiziano

Ahissar, Ehud

Ahissar, Merav

Alards-Tomalin, Doug

Albertazzi, Liliana

Alekseenko, Svetlana

Allard, Rémy

Allison, Robert

Altieri, Nicholas

Andersen, Tobias

Anderson, Barton

Andersson, Linus

Andin, Josefine

Andres, Michael

Andrews, Sally

Andrews, Tim

Anobile, Giovanni

Ansorge, Ulrich

Anstis, Stuart

Apthorp, Deborah

Armann, Regine

Arnold, Derek

Arnoldussen, David

Arrighi, Roberto

Arshamian, Artin

Asgeirsson, Arni

Ashida, Hiroshi

Augusto, Louis

Ayhan, Inci

ayres, Tom

Azzopardi, Paul

Bach, Michael

Backus, Benjamin

Badcock, David

Bae, Gi Yeul

Baker, Daniel

Ballesteros, Soledad

Barraclough, Nick

Barratt, Daniel

Barrett, Brendan

Barrett, Frederick

Bar-Tal, Yoram

Barutchu, Ayla

Bauer, Clemens C. C.

Baumgartner, Elisabeth

Baus, Cristina

Bayliss, Andrew

Baziyan, Boris

Beanland, Vanessa

Becchio, Christina

Beck, Melissa

Belopolsky, Artem

Belopolsky, Victor

Bennetts, Rachel

Bensmaia, Sliman

Benton, Chris

Berman, Marc

Bertamini, Marco

Bertels, Julie

Bex, Peter

Biederman, Irving

Billington, Jac

Billino, Jutta

Bimler, David

Bindemann, Markus

Binetti, Nicola

Bingham, Geoffrey

Birmingham, Elina

Birngruber, Teresa

Blake, Randolph

Blakeslee, Barbara

Bobier, Bill

Boeckler, Anne

Boer, Erwin

Boesveldt, Sanne

Bompas, Aline

Bond, Alan

Bondar, Igor

Bos, Jelte E.

Bosten, Jenny

Bottini, Gabriella

Bottomley, Paul

Bouvet, Lucie

Bowman, Adrian

Brady, Nuala

Braithwaite, Jason

Brascamp, Jan

Braun, Christoph

Braun, Niclas

Brenner, Eli

Bressan, Paola

Brill, Michael

Brockmole, James

Bronstad, Matthew

Brooks, Kevin

Brooks, Joseph

Bruce, Neil

Brugger, Peter

Bubl, Emanuel

Buettner, Andrea

Burke, Darren

Burmester, Alex

Burr, David

Burt, Mike

Burton, Mike

Butterworth, Brian

Cabe, Patrick

Cacciari, Cristina

Caclin, Anne

Cai, zhenguang

Calvillo, Dustin

Candy, Rowan

Cao, Dingcai

Carbon, Claus-Christian

Cardello, Armand

Cardinalli, Lucilla

Carmichael, Duncan

Carvalho, Felipe

Casati, Roberto

Cass, John

Castronovo, Julie

Cattaneo, Zaira

Catteeuw, Peter

Cave, Kyle

Chamberlain, Rebecca

Chaparro, Barbara

Chen, Hui

Chesney, Dana

Chiou, Rocco

Cho, Yang

Choo, Heeyoung

Cicchini, Marco

Claesson, Anna-Sara

Clery, Stephane

Coetzee, Vinet

Cohen, Dale

Cohen, Helen

Cole, Shana

Colizoli, Olympia

Collins, Thérèse

Cometto-Muniz, Enrique

Conway, Bevil

Cormack, Lawrence

Cornelis, Marilyn

Corrow, Sherryse

Craig, James

Crognale, Michael

croijmans, Ilja

crookes, kate

Croy, Ilona

Cutini, Simone

Cutting, James

da Pos, Osvaldo

Dalmaso, Mario

Dana, Kristin

D'Anselmo, Anita

Daunizeau, Jean

Davidoff, Jules

Davies, Leon

Davies-Thompson, Jodie

Davis, Tehran

Dawel, Amy

de Brouwer, Anouk

de Hevia, Lola

de Lussanet, Marc

de Weert, Charles

DeCesari, Andrea

deFockert, Jan

Delplanque, Sylvain

Demareva, Valeriia

Demeyere, Nele

Denham, Sue

Deroy, Ophelia

Deutsch, Diana

de-Wit, Lee

Di Muro, Fabrizio

Diaz, Gabriel

Dijkerman, H.

Dils, Alexia

Ditye, Thomas

Dobs, Katharina

Doerschner, Katja

Domini, Fulvio

Donohue, Sarah

Dorr, Michael

Dresp-Langley, Birgitta

Drewing, Knut

Drieghe, Denis

Du, Schichuan

Duke, Phillip

Dunkley, Ben

Dunning, David

Dupoux, Emmanuel

Durant, Szonya

Durgin, Frank

Dux, Paul

Dyre, Brian

Dzhelyova, Milena

Eckstein, Miguel

Edmonds, Andrew

Ehrsson, Henrik

Eisenbarth, Hedwig

Ekroll, Vebjørn

Elze, Tobias

Ericson, Justin

Erkelens, Casper

Eskew, Rhea

Estudillo, Alejandro

Evans, Samuel

Eves, Frank

Ewing, Louise

Fabbri, Marco

Fang, Fang

Farnè, Alessandro

Fekete, Tomer

Felnhofer, Anna

Fennell, John

Ferdenzi, Camille

Firestone, Chaz

Fiser, Josef

Fleming, Roland

Forde, Ciaran

Foster, Simon

Foulke, Andrew

Franchak, John

Franklin, Anna

Franklin, Nancy

Freiherr, Jessica

Frissen, Ilja

Frowd, Charlie

Froyen, Vicky

Fu, Xiaolan

Fujioka, Takako

Gabay, Shai

Gaby, Jessica

Galmonte, Alessandra

Garcia-Perez, Miguel

Gaskell, Sarah

Geniole, Shawn

Gentaz, Edouard

Georgeson, Mark

Gepshtein, Sergei

Gerbino, Walter

Getz, Laura

Gheorghiu, Elena

Ghuman, Avniel

Giacco, Grazia

Gibson, Gregory

Gilbert, Avery

Gilchrist, Alan

Gilchrist, Iain

Gillam, Barbara

Glennerster, Andrew

Gockel, Hedwig

Gold, Jason

Goldreich, Daniel

Goodale, Melvyn

Gori, Simone

Goutcher, Ross

Graf, Erich

Graham, Daniel

Grainger, Jonathan

Grassi, Massimo

Gray, Rob

Gray, Katie

Greenlee, Mark

Griffin, Lewis

Gross, E. Blair

Grove, Philip

Guest, Steve

Guidi, Stefano

Haigh, Sarah

Halberstadt, Jamin

Halko, Mark

Halper, Fred

Hamilton Fletcher, Giles

Hammett, Stephen

Hancock, Peter

Hannula, Deborah

Hansen, Thorsten

Harris, Laurence

Harrison, Neil

Hartcher-O'Brien, Jess

Hartmann, Matthias

Hasegawa, Yoko

Havelka, Jelena

Hayes, John

Hayward, Vincent

Hayward, William

He, Zijiang

Heed, Tobias

Hein, Elisabeth

Heinrich, Sven

Heller, Morton

Helton, Deak

Henik, Avishai

Hermens, Frouke

Heron, James

Herzmann, Grit

Herzog, Michael

Hesslinger, Vera

Hibbard, Paul

Higham, James

Hill, Harold

Hillstrom, Anne

Hirai, Masahiro

Hiris, Eric

Hirt, Ed

Hoeschele, Marisa

Holcombe, Alex

Hole, Graham

Holmqvist, Kenneth

Holt, Lori

Holten, Vivian

Hommel, Bernhard

Honekopp, Johannes

Hong, Sang

Hoogenhout, Michelle

Hopia, Anu

Horowitz, Todd

Horry, ruth

Hou, Fang

Hout, Michael

Howard, Christina

Howarth, Peter

Hsiao, Janet

Hsu, Li-Chuan

Huber, Stefan

Hung, Shao-Chin

Hutchinson, Claire

Ide, Masakazu

Ilg, Uwe

Ipser, Alberta

Issanchou, Sylvie

Iversen, Wiebke

Jack, Rachael

Jackson, Philip

Jaekl, Philip

James, Thomas

Jansen, Brenda

Javadi, Amir-Homayoun

Jeffery, Linda

Jenkins, Rob

Jennings, Ben

Jensen, Karin

Jiang, Fang

Johnston, Alan

Johnston, Patrick

Jolij, Jacob

Jones, Lynette

Jones, Scott

Jones, Alex

Jones, Benedict

Jones, Luke

Juran, Stephanie

Kamachi, Miyuki

Kaneko, Sae

Kaufman, Lloyd

Kaufmann, Juergen

Kawabe, Takahiro

Kay, Leslie

Keller, Peter

Kelley, Laura

Kelly, David

Kennedy, John

Kerzel, Dirk

Khuu, Sieu

Kibbe, Melissa

Kihara, Ken

Kim, Sungshin

Kingdom, Frederick

Kirby, Anne

Kirtley, Clare

Kitada, Ryo

Kitaoka, Akiyoshi

Kitazaki, Michiteru

Klepousniotou, Ekaterini

Klink, Chris

Kloth, Nadine

Knops, Andre

Koenderink, Jan

Kogo, Naoki

Koning, Arno

Konyshev, Vladimir

Kornmeier, Juergen

Krajcsi, Attila

Kramer, Robin

Krampe, Ralf

Krause, Florian

Kristjánsson, Arni

Kritikos, Ada

Kuai, Shuguang

Kubilius, Jonas

Kubovy, Michael

Kuchinke, Lars

Kuhn, Gustav

Kuhn, Gustav

Kuling, Irene

Kumar, Vijay

Kuroki, Scinob

Kwon, MiYoung

Lacey, Simon

Lacquaniti, Francesco

Lages, Martin

Laming, Donald

Lamm, Claus

Lamy, Dominique

Landis, Basile

Landwehr, Klaus

Lao, Junpeng

Lappe, Markus

Lappin, Joseph

Latanov, Alexandr

Latinus, Marianne

Lawson, Rebecca

Le Corre, Francois

Leder, Helmut

Lee, Kang

Legge, Gordon

Leopold, David

Leth-Steensen, Craig

Levin, Daniel

Levitan, Carmel

Lewis, Michael

Li, Qi

Li, Zhi

Libertus, Melissa

Linares, Daniel

Lingelbach, Bernd

Linkenauger, Sally

Linster, Christiane

Lisi, Matteo

Liston, dorion

Little, Anthony

Little, Daniel

Liu, Jianli

Liu, Taosheng

Liu, Zili

Liuzza, Marco

Lleras, Allajandro

Lloyd, Donna

Locke, John

Logvinenko, Alexander

Longo, Matthew

López-Moliner, Joan

Lorteije, Jeannette

Loschky, Lester

Lu, Hongjing

Lu, Zhong-Lin

Lubeck, Astrid

Lucchina, Laurie

Lundström, Johan

Lupiáñez, Juan

Lynott, Dermot

Macdonald, Rachel

Machilsen, Bart

Macknik, Stephen

MacLin, Kimberly

MacLin, Otto

Maddess, Ted

Maiello, Guido

Majid, Asifa

Mallot, Hanspeter

Maloney, Laurence

Mamassian, Pascal

Mareschal, Isabelle

Marghetis, Tyler

Markovic, Slobodan

Marks, Lawrence

Marmolejo-Ramos, Fernando

Martin, Tim

Martín-Arévalo, Elisa

Martinez-Conde, Susana

Maruya, Kazushi

Marzi, Tessa

Mather, George

Mathes, Birgit

Matsumiya, Kazumichi

Matthews, Nestor

Ma-Wyatt, Anna

McAnany, Jason

McGlone, Frances

McGraw, Paul

McKeown, Denis

McManus, Chris

Meese, Tim

Menneer, Tamaryn

Mens, Lucas

Mestre, Daniel

Mesz, Bruno

Michael, George

Mikellidou, Kyriaki

Millar, Susanna

Milne, Andrew

Mitchell, Derek

Mitsudo, Mitsu

Mizokami, Yoko

Mohr, Christine

Möller, Korbinian

Monaghan, Padraic

Montoro, Pedro

Morash, Valerie

Morgan, Michael

Morikawa, Kazunori

Morrin, Maureen

Morrison, India

Motoyoshi, Isamu

Moulson, Margaret

Mudrik, Liad

Mulder, Max

Munar, Enric

Munzert, Jörn

Murphy, Dana

Murray, Richard

Muth, Claudia

Mylonas, Dimitris

Naber, Marnix

Nakabayashi, Kazuyo

Nakayama, Mariko

Nardini, Marco

Nascimento, Sergio

Nasiopoulos, Eleni

Nater, Urs

Navarra, Jordi

Naveteur, Janick

Navon, David

Nawrot, Elizabeth

Nefs, Harold

New, Joshua

Newell, Fiona

Newport, Roger

Ni, Rui

Niehorster, Diederick

Nikolić, Danko

Ninio, Jacques

Noens, Ilse

Nordin, Steven

Norman, J. Farley

Noyes, Eilidh

Nummenmaa, Lauri

Nuthmann, Antje

Odic, Darko

Okamoto, Shogo

Olkkonen, Maria

Olsson, Mats

Oman, Charles

Ono, Hiroshi

Ono, Takahiro

Orgs, Guido

Otsuka, Yumiko

Overvliet, Krista

Paffen, Chris

Palmer, Stephen

Palmisano, Stephen

Papesh, Megan

Paramei, Galina

Parise, Cesare

Pariyadath, Vani

Parma, Valentina

Parncutt, Richard

Parry, Neil

Pascalis, Olivier

Pasqualotto, Achille

Pastukhov, Alexander

Pathman, Thanujeni

Patro, Katarzyna

Patterson, Michael

Patterson, Roy

Paul, Liz

Pause, Bettina

Pavlova, Marina

Pearson, Joel

Pecher, Diane

Peirce, Jonathan

Pelah, Adar

Pepperell, Robert

Perea, Manuel

Petrini, Karin

Peykarjou, Stefanie

Phillips, Flip

Philpott, Carl

Pia, Lorenzo

Piano, Marianne

Piech, Richard

Pilz, Karin

Pinto, Jayant

Piwek, Lukasz

Plaisier, Myrthe

Podladchikova, Lubov

Pollick, Frank

Pomerantz, James

Pomplun, Marc

Powell, Georgina

Pressnitzer, Daniel

Priftis, Konstantinos

Prince, Melissa

Protonotarios, Manos

Quek, Genevieve

Quené, Hugo

Raymond, Jane

Raynham, Peter

Redies, Christoph

Reeves, Adam

Regenbogen, Christina

Reingold, Eyal

Renier, Laurent

Rensink, Ronald

Richie, Kay

Ricker, Timothy

Riggs, Kevin

Riley, John

Rivolta, Davide

Roberts, Brian

Robinson, Alan

Robinson, Chris

Röder, Susanne

Rodway, Paul

Rogers, Brian

Romano, Daniele

Roseboom, Warrick

Rossion, Bruno

Rothen, Nicolas

Roudaia, Eugenie

Rousselet, Guillaume

Rozhkova, Galina

Rudd, Michael

Rule, Nicholas

Russell, Richard

Saal, Hannes

Salomon, Roy

Sathian, Krish

Saxton, Tamsin

Schaefer, Katrin

Schiltz, Christine

Schloss, Karen

Schmid, Alexandra

Schneider, Tobias

Schneider, Keith

Schofield, Andrew

Schultz, Johannes

Schütt, Heiko

Schwark, Jeremy

Sedgwick, Hal

Seno, Takeharu

Seo, Han-Seok

Serrano-Pedraza,  Ignacio

Serrhini, Kamal

Setlur, Vidya

Seubert, Janina

Sheppard, Jeremy

Shofner, William

Silvera, David

Simmons, David

Simons, Daniel

Sinclair, Rolf

Sinding, Charlotte

Singh, Manish

Sinha, Pawan

Skuk, verena

Sloos, Marjoleine

Smart, Leonard

Smilek, Daniel

Smith, Philip

Smith, Tim

Sobel, Kenith

Solomon, Josh

Sorokowska, Agnieszka

Soute, David

Spang, Karoline

Spector, Ferrinne

Speed, Laura

Spehar, Branka

Spence, Charles

Spering, Miriam

Steadman, Philip

Stefan, Bleeck

Stephen, Ian

Stevenson, Richard

Stevenson, Ryan

Stins, John

Storbeck, Justin

Strasburger, Hans

stronks, H. Christiaan

Sugden, Nicole

Suied, Clara

Suk, Hyeon-Jeong

Sun, Hongjin

Sutherland, Clare

Sven, Panis

Swaddle, John

Swami, Viren

Swaminathan, Jayaganesh

Swapp, David

Sweeny, Timothy

Takahashi, Junichi

Takahashi, Kohske

Takarangi, Melanie

Takeshima, Yasuhiro

Tan, Hong

Tanaka, James

Tanaka, Satoshi

Tatler, Benjamin

Taylor, Christopher

te Pas, Susan

Teigen, Karl Halvor

Temperley, David

Thakral, Preston

Thomas, Peter

Thompson, Ben

Thompson, Peter

Thornton, Ian

Thorpe, Simon

Thurman, Steven

Todd, James

Todorovic, Dejan

Tokita, Midori

Tommasi, Luca

Tong, Jonathan

Toppino, Thomas

Towler, Alice

Townsend, James

Tremoulet, Patrice

Trick, Lana

Trojan, Jörg

Tseng, Philip

Tsougras, Costas

Turi, Marco

Tyler, Christopher

Utochkin, Igor

Utz, Sandra

Valian, Virginia

Valsecchi, Matteo

Valuch, Christian

van Beers, Rob

van Buren, Benjamin

van de Weijer, Jeroen

van den Berg, Bert

van den Berg, Ronald

van der Ham, Sabine

Van der Helm, Peter

van der Kooij, Katinka

van der Stigchel, Stefan

van Doorn, Andrea

van Ee, Raymond

van Leeuwen, Cees

Van Lier, Rob

Van Stralen, Haike

van Thriel, Christoph

Vancleef, Kathleen

vanMarle, Kristy

Vannuscorps, Gilles

Varakin, D Alexander

Vassallo, Suzane

Vaziri Pashkam, Maryam

Velasco, Carlos

Veldhuizen, Marga

Vera-Diaz, Fuensanta

Versnel, Huib

Verstraten, Frans

Vierck, Charles

Vincent, Ben

Vishton, Peter

Vishwanath, Dhanraj

Visser, Troy

Vitu, Françoise

Vocks, Silja

Volkova, Ekaterina

Vuoskoski, Jonna

Wade, Nicholas

Wade, Alex

Wagemans, Johan

Wagman, Jeffrey

Wagner, Valentin

Walk, Anne

Walker, Peter

Wallis, Guy

Wallis, Tom

Wang, Ranxiao

Wann, John

Ward, Jamie

Warren, Paul

Warren, William

Watamaniuk, Scott

Watanabe, Junji

Watanabe, Katsumi

Watson, Tamara

Watson, Derek

Watson, Marcus

Watt, Simon

Webster, Michael

Wegener, Detlef

Welchman, Andrew

Wessberg, Johan

Westheimer, Gerald

Whalen, Paul

White, Alex

White, David

Wichmann, Felix

Wiese, Holger

Wijntjes, Maarten

Wilkie, Richard

Wilkins, Arnold

Williams, Lea

Wilson, John

Wilson, Hugh

Windhager, Sonja

Wise, Paul

Withagen, Rob

Witt, Jessica

Wittmann, Marc

Witzel, Christoph

Wolfe, Jeremy

Wyatte, Dean

Xiao, Naiqi

Xu, Buyun

Yamamoto, Kentaro

Yarrow, Kielan

Yau, Jeffrey

Yonas, Albert

Young, Angela

Zaidi, Qasim

Zavagno, Daniele

Zdravkovic, Suncica

Zenkin, Gary

Zhang, Jun-Yun

Zhang, Xilin

Zhao, Mintao

Zhaoping, Li

Zhegallo, Alexander

Zhou, Jiawei

Zopf, Regine

Zylinski, Sarah

